# Addressing Domestic Violence Against Women: An Unfinished Agenda

**DOI:** 10.4103/0970-0218.40871

**Published:** 2008-04

**Authors:** Ravneet Kaur, Suneela Garg

**Affiliations:** Department of Community Medicine, Maulana Azad Medical College, New Delhi - 110 002, India

“Domestic violence is a burden on numerous sectors of the social system and quietly, yet dramatically, affects the development of a nation… batterers cost nations fortunes in terms of law enforcement, health care, lost labor and general progress in development. These costs do not only affect the present generation; what begins as an assault by one person on another, reverberates through the family and the community into the future”. (Zimmerman)(1)

Domestic violence is a global issue reaching across national boundaries as well as socio-economic, cultural, racial and class distinctions. This problem is not only widely dispersed geographically, but its incidence is also extensive, making it a typical and accepted behavior. Domestic violence is wide spread, deeply ingrained and has serious impacts on women's health and well-being. Its continued existence is morally indefensible. Its cost to individuals, to health systems and to society is enormous. Yet no other major problem of public health has been so widely ignored and so little understood.(2)

## What is Domestic Violence?

Domestic violence can be described as the power misused by one adult in a relationship to control another. It is the establishment of control and fear in a relationship through violence and other forms of abuse. This violence can take the form of physical assault, psychological abuse, social abuse, financial abuse, or sexual assault. The frequency of the violence can be on and off, occasional or chronic.

“Domestic violence is not simply an argument. It is a pattern of coercive control that one person exercises over another. Abusers use physical and sexual violence, threats, emotional insults and economic deprivation as a way to dominate their victims and get their way”. (Susan Scheter, Visionary leader in the movement to end family violence)(3)

The Protection of Women from Domestic Violence Act, 2005 says that any act, conduct, omission or commission that harms or injures or has the potential to harm or injure will be considered domestic violence by the law. Even a single act of omission or commission may constitute domestic violence - in other words, women do not have to suffer a prolonged period of abuse before taking recourse to law. The law covers children also.(4) Domestic violence is perpetrated by, and on, both men and women. However, most commonly, the victims are women, especially in our country. Even in the United States, it has been reported that 85% of all violent crime experienced by women are cases of intimate partner violence, compared to 3% of violent crimes experienced by men.(5) Thus, domestic violence in Indian context mostly refers to domestic violence against women.

## Problem Statement

Domestic violence is the most common form of violence against women. It affects women across the life span from sex selective abortion of female fetuses to forced suicide and abuse, and is evident, to some degree, in every society in the world.

The World Health Organization reports that the proportion of women who had ever experienced physical or sexual violence or both by an intimate partner ranged from 15% to 71%, with the majority between 29% and 62%.(2)

India's National Family Health Survey-III, carried out in 29 states during 2005-06, has found that a substantial proportion of married women have been physically or sexually abused by their husbands at some time in their lives. The survey indicated that, nationwide, 37.2% of women “experienced violence” after marriage. Bihar was found to be the most violent, with the abuse rate against married women being as high as 59%. Strangely, 63% of these incidents were reported from urban families rather than the state's most backward villages. It was followed by Madhya Pradesh (45.8%), Rajasthan (46.3%), Manipur (43.9%), Uttar Pradesh (42.4%), Tamil Nadu (41.9%) and West Bengal (40.3%).(6)

The trend of violence against women was recently highlighted by the India's National Crime Records Bureau (NCRB) which stated that while in 2000, an average of 125 women faced domestic violence every day, the figure stood at 160 in 2005.(7)

A recent United Nation Population Fund report also revealed that around two-thirds of married women in India were victims of domestic violence. Violence in India kills and disables as many women between the ages of 15 and 44 years as cancer and its toll on women's health surpasses that of traffic accidents and malaria combined.(8)

Even these alarming figures are likely to be significantly under estimated given that violence within families continues to be a taboo subject in both industrialized and industrializing countries.

## What Leads to Domestic Violence?

Domestic violence against women is an age old phenomenon. Women were always considered weak, vulnerable and in a position to be exploited. Violence has long been accepted as something that happens to women. Cultural mores, religious practices, economic and political conditions may set the precedence for initiating and perpetuating domestic violence, but ultimately committing an act of violence is a choice that the individual makes out of a range of options. Although one cannot underestimate the importance of macro system-level forces (such as cultural and social norms) in the etiology of gender-based violence within any country, including India, individual-level variables (such as observing violence between one's parents while growing up, absent or rejecting father, delinquent peer associations) also play important roles in the development of such violence. The gender imbalance in domestic violence is partly related to differences in physical strength and size. Moreover, women are socialized into their gender roles in different societies throughout the world. In societies with a patriarchal power structure and with rigid gender roles, women are often poorly equipped to protect themselves if their partners become violent. However, much of the disparity relates to how men-dependence and fearfulness amount to a cultural disarmament. Husbands who batter wives typically feel that they are exercising a right, maintaining good order in the family and punishing their wives' delinquency - especially the wives' failure to keep their proper place.(9)

## Domestic Violence and its Health Implications

Violence not only causes physical injury, it also undermines the social, economic, psychological, spiritual and emotional well being of the victim, the perpetrator and the society as a whole. Domestic violence is a major contributor to the ill health of women.

It has serious consequences on women's mental and physical health, including their reproductive and sexual health. These include injuries, gynecological problems, temporary or permanent disabilities, depression and suicide, amongst others.

“Many forms of verbal and psychological abuse appear relatively harmless at first, but expand and grow more menacing over time, sometimes gradually and subtly. As victims adapt to abusive behavior, the verbal or psychological tactics can gain a strong ‘foothold’ in victims' minds, making it difficult for them to recognize the severity of the abuse over time.” (Witness Justice, MA, USA)(3)

These physical and mental health outcomes have social and emotional sequelae for the individual, the family, the community and the society at large.

Over both the short term and long term, women's physical injuries and mental trouble either interrupts, or ends, their educational and career paths leading to poverty and economic dependence. Family life gets disrupted which has a significant effect on children, including poverty (if divorce or separation occurs) and a loss of faith and trust in the institution of the family. These sequelae not only affect the quality of life of individuals and communities, but also have long-term effects on social order and cohesion.(9)

In India, one incident of violence translates into the women losing seven working days. In the United States, total loss adds up to 12.6 billion dollars annually and Australia loses 6.3 billion dollars per year.(8)

The physical health consequences of domestic violence are often obscure, indirect and emerge over the long term. For example, women who were subject to violent attacks during childhood are bothered by menstrual problems and irritable bowel syndrome in later life.(9)

## Domestic Violence and Reproductive Health

There is enough evidence to support that higher reproductive morbidity is seen among women experiencing domestic violence. Studies conducted in North India have shown elevated odd's ratio of gynecological symptoms, while comparing women with husbands reporting no domestic violence and women who experienced physical and sexual violence. It may be attributed to the fact that abusive men were more likely to engage in extra marital sex and acquire STDs, there by placing their wives at risk of acquiring STDs. There was also lesser condom use reported among such men.(10)

These make women more susceptible to HIV infection, and the fear of violent male reactions, physical and psychological, prevents many women from trying to find out more about it, discourages them from getting tested and stops them from getting treatment.(7)

Studies in the northern state of Uttar Pradesh have also shown that unplanned pregnancies are significantly more common among wives of abusive men (OR = 2.62)(11). Besides this, research has shown that battered women are subject to twice the risk of miscarriage and four times the risk of having a baby that is below average weight. In some places, violence also accounts for a sizeable portion of maternal deaths.(9) Reproductive health care that incorporates domestic violence support services is needed to meet the special needs of abused women.

## Psychological and Emotional Violence

Psychological and emotional violence covers “repeated verbal abuse, harassment, confinement and deprivation of physical, financial and personal resources”.

Quantifying psychological abuse is extremely difficult, and very few studies have been conducted to establish prevalence rates of this type of violence. Qualitative studies that have been undertaken conclude that it is just as damaging to one's health to be continuously psychologically abused as it is to be physically abused. Undermining an individual's sense of self esteem can have serious mental and physical health consequences and has been identified as a major reason for suicide. For some women, the incessant insults and tyrannies which constitute emotional abuse may be more painful than the physical attacks because they effectively undermine women's security and self-confidence.(9)

Violence against women has a far deeper impact than the immediate harm caused. It has devastating consequences for the women who experience it and a traumatic effect on those who witness it, particularly children.(2)

## Impact of Domestic Violence on Children

Children who witness domestic violence may develop serious emotional, behavioral, developmental or academic problems.

As they develop, children and teens who grow up with domestic violence in the household are:

- more likely to use violence at school or community in response to perceived threats
- more likely to attempt suicide
- more likely to use drugs
- more likely to commit crimes, especially sexual assault
- more likely to use violence to enhance their reputation and self esteem
- more likely to become abusers in later life

## Why Do Women Stay?

Economic dependence has been found to be the central reason. Without the ability to sustain themselves economically, women are forced to stay in abusive relationships and are not able to be free from violence. Due to deep-rooted values and culture, women do not prefer to adopt the option of separation or divorce. They also fear the consequences of reporting violence and declare an unwillingness to subject themselves to the shame of being identified as battered women. Lack of information about alternatives also forces women to suffer silently within the four walls of their homes.(3) Some women may believe that they deserve the beatings because of some wrong action on their part. Other women refrain from speaking about the abuse because they fear that their partner will further harm them in reprisal for revealing family secrets, or they may be ashamed of their situation.

Violence against women is a violation of basic human rights. It is shameful for the states that fail to prevent it and societies that tolerate and in fact perpetuate it. It must be eliminated through political will, and by legal and civil action in all sectors of society.

## Addressing Domestic Violence

An effective response to violence must be multi-sectoral; addressing the immediate practical needs of women experiencing abuse; providing long-term follow up and assistance; and focusing on changing those cultural norms, attitudes and legal provisions that promote the acceptance of and even encourage violence against women, and undermine women's enjoyment of their full human rights and freedoms.

The health sector has unique potential to deal with violence against women, particularly through reproductive health services, which most women will access at some point in their lives. However, this potential is far from being realized. Few doctors, nurses or other health personnel have the awareness and the training to identify violence as the underlying cause of women's health problems.

The health sector can play a vital role in preventing violence against women, helping to identify abuse early, providing victims with the necessary treatment and referring women to appropriate care. Health services must be places where women feel safe, are treated with respect, are not stigmatized, and where they can receive quality, informed support. A comprehensive health sector response to the problem is needed, in particular addressing the reluctance of abused women to seek help.(2)

## Role of Public Health Personnel

Domestic violence against women has been identified as a public health priority. Public health personnel can play a vital role in addressing this issue.

Since violence against women is both a consequence and a cause of gender inequality, primary prevention programs that address gender inequality and tackle the root causes of violence are all essential. Public health workers have a responsibility to build awareness by creating and disseminating materials and innovative audio-visual messages, which project a positive image of girl child and women in the society. An integrated media campaign covering electronic, print and film media that portrays domestic violence as unacceptable is the need of the hour. The role of increasing male responsibility to end domestic violence needs to be emphasized.

Programs are required which intend to address battered women's needs, including those that focus on building self-efficacy and livelihood skills. The significance of informal and local community networks should be acknowledged in this regard. The survivors of domestic violence can be involved in program planning and implementation in order to ensure accessibility and effectiveness.(12) Rather than spotlighting women as victims in non negotiable situations, they should be portrayed as agents capable of changing their own lives. The public health experts have a vital role to play in networking with NGOs and voluntary organizations and creation of social support networks.

The public health experts have a potential to train personnel specialized to address the needs of victims of domestic violence. In the field of research, public health personnel can contribute by conducting studies on the ideological and cultural aspects which give rise to and perpetuate the phenomenon of domestic violence. Similarly, the execution and impact of programs must be assessed in order to provide the necessary background for policy-making and planning. However, the health sector must work with all other sectors including education, legal and judicial, and social services.(2)

In January, India implemented its first law aimed at tackling domestic violence (*The Protection of Women from Domestic Violence Act, 2005*) to protect the rights of women who are victims of violence of any kind occurring within the family and to provide for matters connected therewith or incidental thereto. It also defines repeated insults, ridiculing or name-calling, and demonstrations of obsessive possessiveness and jealousy of a partner as domestic violence. The big challenge in front now is to enforce it in true sense.

“A law is as good as its implementability, despite the lofty aspirations. The responses to the enactment are polarized, with one section fearing its misuse by an elite class in metro cities and another segment predicting its futility for the mass of rural women saddled with the yoke of patriarchy to which courts are as yet alien” (Flavia Agnes)(13)

A bill alone will not help in preventing domestic abuse; what is needed is a change in mindsets.

Concerted and co-ordinated multisectoral efforts are key methods of enacting change and responding to domestic violence at local and national levels. The Millennium Development Goal regarding girls' education, gender equality and the empowerment of women reflects the international community's recognition that health, development, and gender equality issues are closely interconnected.

Hence the responses to the problem must be based on integrated approach. The effectiveness of measures and initiatives will depend on coherence and co ordination associated with their design and implementation. The issue of domestic violence must be brought into open and examined as any other preventable health problem, and best remedies available be applied.

## References

[1] Zimmerman C (1994). Plates in a basket will rattle: Domestic violence in Combodia, Phnom Pehn.

[2] WHO (2007). Multi country study on Women's health and domestic violence against women.

[3] (2005). Fact Sheet: Domestic violence: Older women can be victims too. National center on elder abuse.

[5] Rennison CM http://www.ojp.usdoj.gov/bjs/pub/pdf/ipv01.pdf.

[6] Fact Sheet: National Family Health Survey NFHS-III 2005-06. Ministry of Health and Family Welfare.

[7] http://HYPERLINK.

[8] Two- third married Indian women victims of domestic violence.

[9] WHO (2001). Domestic violence: A priority public health issue in western Pacific region. Western Pacific Regional Office.

[10] Stephenson R, Koening MA, Ahmed S (2006). Domestic violence and symptoms of gynecological morbidity among women in North India. Int Fam Plan Perspect.

[11] Martin SL, Kilgallen B, Tsui AO, Maitra K, Singh KK, Kupper LL (1999). Sexual behaviors and Reproductive health outcomes: Associations with wife abuse in India. JAMA.

[12] Burlon B, Duvvury N, Varia N (2000). Justice, Change and Human Rights: International Research and Response to Domestic Violence: Jointly published by International Center for Research on Women and Center for Development and Population Activities.

[13] Agnes F (2005). Domestic violence act - A portal of hope. Combat Law.

